# How Does Climate Change Affect Tomato and Okra Production? Evidence from Nigeria

**DOI:** 10.3390/plants12193477

**Published:** 2023-10-04

**Authors:** Robert Ugochukwu Onyeneke, Fred Fosu Agyarko, Chinenye Judith Onyeneke, Emeka Emmanuel Osuji, Patience Afor Ibeneme, Iman Janghorban Esfahani

**Affiliations:** 1 Department of Agriculture, Alex Ekwueme Federal University Ndufu-Alike, Ikwo 482131, Nigeria; robert.onyeneke@funai.edu.ng (R.U.O.); onyeneke.chinenye@funai.edu.ng (C.J.O.); osujiemeka2@gmail.com (E.E.O.); 2 Institute for Scientific and Technological Information (INSTI), Accra P.O. Box M 32, Ghana; agyarkofosu@gmail.com; 3 Department of Geography, Alex Ekwueme Federal University Ndufu-Alike, Ikwo 482131, Nigeria; patienceibeneme@gmail.com; 4 Glopex Co., Ltd., R & D Center B2065, GeumGang Penterium IX Tower A2801, Dongtancheomdansaneop 1-ro 27, Hwaseong-si 18469, Gyeonggi-do, Republic of Korea

**Keywords:** tomato yield, okra yield, climate, credit, fertilizer, autoregressive distributed lag model

## Abstract

This study examined the impacts of climate change on okra and tomato yields. Fertilizer consumption and credit to the crop sector were considered as covariates in the analysis. Time-series data, spanning a period of 40 years, were obtained from various sources. An autoregressive distributed lag model was applied to analyze short- and long-term impacts of climate change and agricultural inputs on okra and tomato yields. Not all variables were stationary at levels (order zero), but they were all significant at first difference, indicating the presence of cointegration. The Bound’s test F-ratio was statistically significant and implied the presence of long- and short-term relationships among the variables studied. The mean temperatures had negative impacts on okra and tomato yields in both the short and long terms. Credit guaranteed to the crop sector had positive short- and long-term impacts on tomato yield; fertilizer consumption had a negative long-term impact on okra yield. Our study concludes that climate change, particularly rising temperature, impacts herbaceous fruit crop production in Nigeria. Therefore, we recommend that breeding and disseminating climate-smart tomato and okra varieties will help fruit crop farmers respond to rising temperatures.

## 1. Introduction

One of the greatest issues of the 21st century is global climate change. According to a growing body of evidence, climate change is undeniable and occurring at an incredible rate [1,2]. The vast tropical and subtropical areas that make up about two thirds of the African continent are predicted to be adversely impacted by climate change [3,4]. Variations in rainfall patterns and extreme weather occurrences, including recurrent droughts, floods, and windstorms, characterize many of the effects of climate change in these regions. Even more so, rising temperatures, humidity variations, and sunlight availability result in overall low crop productivity [5,6,7]. In Africa, about 70 percent of household farmers who rely heavily on crop production for economic livelihood and survival are severely impacted by the effects of climate change [8,9,10]. Nigeria is currently challenged by climate change, which affects agriculture and other sectors of the economy [11,12].

Rising temperatures, unpredictable rainfall patterns, rising sea levels, flooding, drought and desertification, land degradation, increased frequency of extreme weather events, depleted fresh water supplies, and declining biodiversity are all manifestations of climate change in Nigeria [13,14]. Rainfall has risen in duration and intensity, resulting in significant runoff and flooding events in several areas of Nigeria, and it is predicted that rainfall and temperature variability will continue to rise in decades to come [15,16]. Rising sea levels are predicted to worsen flooding and the submergence of coastal lands as precipitation increases in southern regions [17,18]. Due to a decrease in precipitation and an increase in temperature, droughts have also become a recurring problem in Nigeria and are predicted to persist in Northern Nigeria [19,20,21]. The importance of herbaceous fruit crop (such as okra and tomato) production in Nigeria cannot be undervalued, as this production contributes to the gross domestic product (GDP) in Nigeria and, to a large extent, provides the basic food needs for our teeming population [22]. Many farmers engage in the cultivation of tomato, okra, and other fruit crops, making them home-grown crops and sources of livelihood and upkeep for many families [23]. Tomato, okra, and other herbaceous fruit crops are packed with vital vitamins, minerals, and antioxidants that offer the body numerous significant health advantages [24]. For instance, tomatoes are the main nutritional source of the antioxidant lycopene, which has been associated with a number of health advantages, such as a decreased risk of cancer and heart disease. The nutrients vitamin C, potassium, folate, and vitamin K are also abundant in them. The production of okra in Nigeria has increased due to its high nutritional value, as it provides important sources of protein, vitamin A and C, carbohydrates, calcium, potassium, magnesium, and other minerals, which are often lacking in people’s diets [25,26]. Its medicinal abilities can be seen in the treatment of peptic ulcers and its use as a source of plasma replacement in body fluids [27].

Magnesium is another important nutrient found in okra that is necessary for healthy bodily growth and function [28]. Climate change impacts on fruit crop production have resulted in low quality, quantity, and stunted yields of the crops [29,30]. Thus, any slight variations in weather conditions affect yield negatively. Fruits are typically more vulnerable to weather extremes, such as high temperatures and soil moisture stress, which cause the crops to shrivel and their leaves to turn yellow [31,32]. A significant greenhouse gas, CO_2_, also has an impact on the development and growth of crops, as well as on the prevalence of insect pests and diseases [33].

The cultivation of fruit crops, therefore, is becoming unprofitable due to crop failures, low yields, declining quality, and rising pest and disease issues in climates that are changing [34]. Consequently, the crops are especially vulnerable to the effects of climate change because they lose a lot of water through evapotranspiration in hot, dry climates, creating salty deposits around the roots that prevent the crops from absorbing water and, thus, lower their yields and output [30].

Some theoretical and empirical studies have focused on investigating climate impacts on cereal crops, such as rice, maize, wheat, and sorghum [35,36,37,38], and tuber crops [39,40], such as cassava. There is growing debate on the impacts of climate change on agriculture, and several attempts have been made to model climate change and agricultural production interaction. Many of these studies have suggested that climate change impacts Nigeria’s agriculture negatively [41,42,43,44,45,46].

However, with regards to changing climate, the importance of fruits to food and nutrition security, the vulnerability of herbaceous fruit crops to climate vagaries, and the importance of other agricultural production inputs (credit and fertilizer) in herbaceous fruit crop production in Africa’s most populous nation—Nigeria—research studies are needed to understand the extent to which herbaceous fruit crops would be affected by changes in temperature and precipitation. This will contribute to the design and development of climate-resilient strategies for the herbaceous fruit crop sub-sector, the attainment of the Sustainable Development Goals, the actualization of the country’s agricultural policy, and the achievement of the ambitions of the Nationally Determined Contribution, as well as other climate commitments.

Several studies have been undertaken to understand the impacts of climate change on crop production in Nigeria [45,47,48,49,50,51,52,53,54,55,56,57,58,59,60,61,62,63,64,65,66]. Meanwhile, there is compelling evidence suggesting that inputs also affect production over time [45,58]. Overall, most of the previous studies support the view that climate variables and agricultural inputs impact crop production over time. However, some of the studies on climate change and crop production in Nigeria have not given adequate attention to the effects of climate variables and crop inputs on crop production (particularly in fruit crops) over time, and this may limit our understanding on the overall change happening in the country’s fruit farming sector. Moreover, information on the impacts of climate change on herbaceous fruit crop yield in Africa is limited and not well understood. Therefore, to sustain herbaceous fruit crop production under a changing climate, the impact of the changing climate, as well as the availability of agricultural inputs (such as fertilizer and credit), on herbaceous fruit crop production should be evaluated. Thus, researchers have paid much attention to the impact of climate change and agricultural inputs on cereal, tuber, and root crop production (see [45,47,48,49,50,51,52,53,54,55,56,57,58,59,60,61,62,63,64,65,66]), with limited attention to herbaceous fruit crops, which also contribute significantly to the food, nutrition, and diet needs of the populace. Therefore, there is a paucity of information on both country-specific and continent-specific analyses regarding the long-term impacts of climate change on herbaceous fruit production. This issue motivated the need for this study. The findings of this study may help decision makers in Nigeria design and implement appropriate climate-resilient strategies targeted at herbaceous fruit crop production under the current changing climatic conditions in the country. Therefore, the main objective of this study was to analyze the impacts of climate change on herbaceous fruit crop (tomato and okra) production in Africa’s most populous country—Nigeria.

## 2. Materials and Methods

### 2.1. Data Source

This paper used time-series data (1981–2020) obtained from four databases—the Central Bank of Nigeria Statistical Bulletin, FAOSTAT, World Development Indicators, and the World Bank Climate Change Knowledge Portal. We used data obtained from the Central Bank of Nigeria 2020 Statistical Bulletin, FAOSTAT, and World Bank Climate Change Knowledge Portal. Specifically, tomato and okra yield statistics were obtained from the FAOSTAT database, fertilizer consumption data were obtained from the World Development Indicators database, temperature and rainfall statistics were obtained from World Bank Climate Change Knowledge Portal, while credit guaranteed to the crop sector data were obtained from the Central Bank of Nigeria Statistical Bulletin. The details of the data and sources are presented in Table 1. In order to control for inflation in our dataset, official exchange rate statistics were obtained from the Central Bank of Nigeria Statistical Bulletin, and we used the figures to convert Naira values of the credit guaranteed to the crop sector to United States Dollars.

### 2.2. Data Analysis

We used the autoregressive distributed lag (ARDL) model to analyze the impacts of climate change and production inputs on okra and tomato yield in Nigeria. The ARDL has the ability to model both the short- and long-term impacts between dependent variable(s) and independent variables [45,46,58]. The Augmented Dickey Fuller (ADF) and the Phillips–Perron tests were employed to determine the existence of unit roots in our dataset. Furthermore, the White’s test, Ramsey RESET test, variance inflation factor, cumulative sum, and cumulative sum square were employed to test for heteroskedasticity, presence of omission variable, multicollinearity, and parameter stability of our model, respectively. The dependent variables of this study were okra and tomato yields, while the independent variables were mean temperature, total rainfall, fertilizer consumption, and credit guaranteed to the crop sector.

We applied the popular autoregressive distributed lag (ARDL) approach by Pesaran et al. [71] to model the impacts of climate change and agricultural inputs on herbaceous fruit crop production in Nigeria. This model is considered as the best econometric method compared to others in a case when the variables are stationary at I(0) or integrated of order I(1) [72]. Based on the study objectives, it is a better model than others because it produces short- and long-term impacts of independent variables (temperature, rainfall, fertilizer, and credit) on okra and tomato production in Nigeria. The ARDL model is most appropriate for generating short- and long-run estimates of the independent variables over time and uses the ordinary least square (OLS) approach for cointegration between variables [73]. This model affords flexibility about the order of integration of the variables and it is suitable for predictors in the model, which is I(0), I(1), or mutually cointegrated [72,74,75].

The implicit model of the autoregressive distributed lag framework used in this study is stated as follows:

(1) Yi=f(X1,X2,X3,X4,e)

where Yi = herbaceous fruit crops yield, X1 = mean temperature (°C), X2 = total rainfall (mm), X3 = fertilizer consumption (kilograms per hectare of arable land), X4 = total credit guaranteed to the crop sector (USD), e = error term, and i = 1 (okra yield), 2 (tomato yield).

### 2.3. Summary Statistics

Table 2 presents the results of the descriptive statistics of the dependent and independent variables of this study. The Table shows that the minimum value of okra yield was 8735/hg/ha, the maximum value was 29,962 hg/ha, while the average was 21,630.63 hg/ha. The standard deviation (5797.07 hg/ha) of the okra yield was huge, indicating that okra yield in the country is not normally distributed (fairly normal). This argument is further supported by the coefficient of the skewness, which was −0.86. Hence, we can say that the distribution of okra production in Nigeria is moderately skewed to the left. Since okra production is negatively skewed, it implies that most of the yields recorded across the years studied were close to the maximum value (29,962 hg/ha) with few close to the minimum value (8735 hg/ha).

The average tomato yield was 76,881.20 hg/ha, the minimum yield under for the studied period was 37,208 hg/ha, while the maximum yield was 103,455 hg/ha. The coefficient of skewness (−0.35) of tomato yield showed that most of the yields recorded across the years studied were close to the maximum value, with few close to the minimum value. Average temperature was 27.17 °C, while the average total rainfall was 1137.11 mm. Average fertilizer consumption was 10.04 kg per hectare of arable land, while average total credit guaranteed to the crop sector was USD 17,196.85.

## 3. Results

### 3.1. Testing for Multicollinearity

Table 3 presents the result of the variance inflation factor (VIF) for determining the presence of multicollinearity among the independent variables. The Table shows that the VIF for each independent variable is less than 5, which indicates the absence of multicollinearity. Onyeneke et al. [76], Onyeneke et al. [77], Onyeneke et al. [78], Chidiebere-Mark et al. [79], and Emenekwe et al. [58] used VIF of 5 as an acceptable limit of multicollinearity. Hence, any VIF higher than 5 indicates the presence of multicollinearity among the variables.

### 3.2. Unit Roots Test

Table 4 presents the stationarity test results. We investigated the stationarity of our variables by conducting two unit root tests—the Augmented Dickey–Fuller test and Phillips–Perron test. Table 4 shows that, under the Augmented Dickey–Fuller test, all the variables with the exception of tomato yield were not stationary when integrated at order zero (I(0)). However, all the variables were stationary at the first difference (i.e., these variables were integrated at order one). Under the Philips–Perron test, the Table shows that okra yield, fertilizer consumption, and total credit guaranteed to the crop sector were not stationary when integrated at order zero. However, tomato yield, mean temperature, and total rainfall were all stationary at the first difference under the Phillips–Perron test. Since all the variables were stationary at first difference under both the Augmented Dickey–Fuller and the Phillips–Perron models, it is necessary to employ ARDL model to further analyze the impact of the independent variables on the dependent variables.

### 3.3. Cumulative Sum and Cumulative Sum of Squares Plot

**Figure 1:**
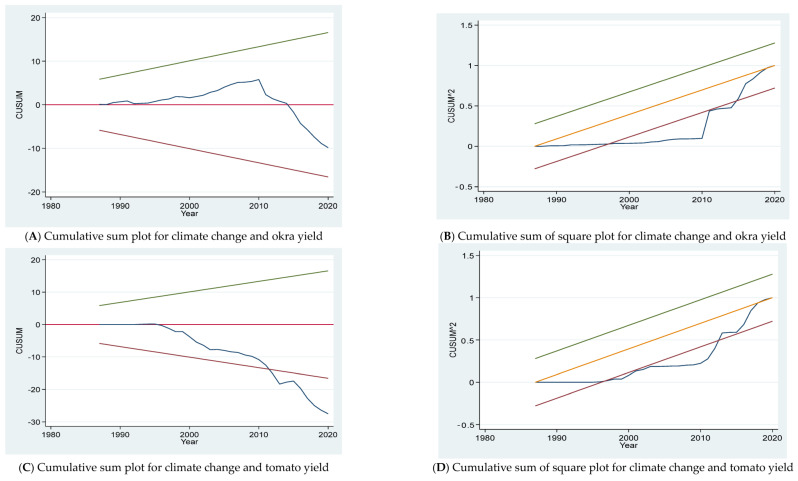
Cumulative sum and cumulative sum of square tests plots for parameter stability for okra and tomato yield models.

We used the cumulative sum (CUSUM) and cumulative sum of squares (CUSUMSQ) tests recommended by Pesaran and Shin [80] and Pesaran et al. [71] to test the structural stability of our model. Figure 1 presents the results of the cumulative sum (CUSUM) and cumulative sum of squares (CUSUMSQ) tests of okra and tomato yields versus the independent variables. The figure shows that the econometric models are structurally stable. This again confirms the appropriateness of the autoregressive distributed lag model used in this paper.

### 3.4. Cointegration Test

We used the Ramsey RESET test to examine whether the model has an omitted variable problem or not. The Ramsey RESET test result is presented in Table 5. The table shows that both models (okra and tomato yield models) were not statistically significant, indicating that these models are free from omitted variable problems. The White’s test was used to test for homoskedasticity in the econometric model. The result is presented in Table 5. The White’s test results for both the okra yield and the tomato yield models were not statistically significant. This implies that there is an absence of heteroskedasticity in the okra and tomato yield models.

We used the Bounds test to determine whether there is a long-run relationship between the dependent variables (okra and tomato yield) and independent variables (mean temperature, precipitation, fertilizer consumption, and total credit guaranteed to the crop sector). The result is also presented in Table 5. The calculated F-ratio for okra yield (5.077) and tomato yield (3.504) were statistically significant at 1% and 10% levels of probability, respectively. We used the Kripfganz and Schneider [81] critical values and approximate p-values produced by STATA 17 software. This result implies that there are long-run relationships between okra and tomato yields and the independent variables. Onyeneke et al. [45] also found a long-run relationship between cereal and root and tuber crop production (rice, millet, sorghum, and maize, cassava, and yam) and temperature, rainfall, land, fertilizer, and credit in Nigeria. Chidiebere-Mark et al. [79] found a similar result in Africa in a study where the impacts of agricultural production, renewable energy, and foreign investment on greenhouse gas emissions were explored.

### 3.5. Short- and Long-Run Impacts of Climate Change on Fruit Crops Yield

Table 6 presents the ARDL estimates of the impact of climate change on herbaceous fruit crop yield. The table shows that mean temperature negatively and significantly affected okra and tomato yields in the long run. Under okra yield, the estimate recorded for mean temperature was −28.96. This implies that, all other things being equal, a 1% increase in the mean temperature would decrease okra yield by approximately 29%. Moreover, the estimate of mean temperature under tomato yield was −35.81. This estimate (−35.81) statistically implies that a 1% increase in the mean temperature would decrease tomato yield by approximately 35.8% in the long run. Although our analysis has shown that increasing temperature would decrease both okra and tomato yields in the long run, tomato may suffer a greater decline in yield as compared to okra. Thus, rising temperature would negatively impact on tomato yield more than okra yield.

Fertilizer consumption negatively and significantly affected okra yield in the long run. The coefficient was −0.48, implying that a 1% increase in fertilizer consumption would decrease okra yield by approximately 0.48% in the long run.

Total credit guaranteed to the crop sector positively but insignificantly affected okra yield. For the tomato yield model, the estimate of total credit guaranteed to the crop sector was positive (0.15) and statistically significant at 5%. Thus, in the long run, a 1% increase in the total credit guaranteed to the crop sector would increase tomato yield by 0.15%, ceteris paribus.

We now focus on interpreting the results of the short-run estimates. Mean temperature estimate was statistically significant under both models (okra and tomato yield). From Table 6, mean temperature estimate under the okra yield model was negative and statistically significant at 5%. This indicates that a 1% percent increase in total rainfall would lead to a 6.9% decrease in okra yield and 6.67% in tomato yield in the short run.

The short-run and the long-run effects of the total credit guaranteed to the crop sector on crop yields (okra and tomato) are the same. The estimate of total credit guaranteed to the crop sector under the okra yield model was positive but not statistically significant, both in the long run and short run. However, the total credit guaranteed to the crop sector was positively and significantly related tomato yield both in the, short and long runs. Therefore, total credit guaranteed to the crop sector had a positive significant impact on average tomato yield in the short run. Thus, a 1% increase in credit guaranteed to the crop sector would increase tomato by 0.07% in the short run. 

## 4. Discussion

This study analyzed the effects of climate change (proxied by average temperature and precipitation) and agricultural production inputs (such as credit and fertilizer) on herbaceous fruit crop production (okra and tomato). With regards to the effect of temperature and the fruit crop yields, we found that mean temperature had significant and negative relationships with both crops (okra and tomato) in both the short and the long terms. This suggests that rising temperature significantly decreased herbaceous fruit crop yield in the country. Camejo et al. [82], in their study, observed that the highest temperature for cultivating tomato is between 25 °C and 30 °C in the daytime and 20 °C at night. Thus, any temperature beyond these thresholds will be detrimental to the crop. The effects may lead to decrease in pollen quality, flower abscission, abnormal growth, and reduction in fruit bearing [82]. Our results are in conformity to the study of Hazra et al. [83]. Hazra et al. [83] found that there is a negative relationship between tomato yield and temperature. They further explained that, when tomato crops are exposed to higher temperature (34/19 °C), crop flowering will drop by 34%, thereby decreasing fruit set by 71%. Recently, Vijayakumar and Beena [84], in their study, assessed the impact of temperature difference on the physicochemical properties and yield of tomato. They found that rising temperature inhibits seed germination, retards plant growth, deteriorates fruit quality, alters photosynthesis, reduces dry matter production, causes water loss, causes oxidative stress, and, finally, decreases yield.

On the effect of fertilizer use and herbaceous fruit crop yields in Nigeria, we found that fertilizer application had a negative and significant impact on okra yield in the long run. However, in the short run, the result showed a positive but statistically insignificant relationship with okra yield in Nigeria. Masuku et al. [85] also found a negative relationship between fertilizer use and green pepper production in Swaziland. This result implies that fertilizer application could increase okra production in the short run in the country, but this will come with a significant negative effect in the long run, thereby decreasing the yield of the crop. Muhammad et al. [86] noted that inorganic fertilizer use could increase okra production, but inorganic fertilizers are not environmentally friendly. Continuous and inappropriate use of fertilizer in okra production may lead to soil pollution and degradation, which may negatively affect production in the long run. This result substantiates the finding of Onyeneke et al. [45], who found negative long-term impacts of nitrogen, potash, and phosphate use per unit land area on outputs of cereal and tuber crops in Nigeria. Therefore, efficient use of fertilizer is needed to attain appropriate nutrient budget level [87], particularly for herbaceous fruit crop farming. In fact, Makinde et al. [26], Shampazuraini et al. [26], and Purbajanti et al. [88], in their studies, found that inorganic fertilizer combined with organic fertilizer in okra production is better in terms of productivity/yield. Hence, combination of organic and inorganic fertilizer could be recommended for effective production, enhanced climate resilience, and mitigation.

We also examined the impact of credit on herbaceous fruit crop yields. Our result shows that credit increased tomato production. Our finding shows that credit had a positive impact on okra and tomato production, both in the short run and long run. While the effect was statistically significant for tomato production, it was not for okra production, both in the short and long terms. Our result indicates that credit can boost tomato production in the long run. Credit increases agricultural production in the short run [89,90]. For instance, Mariyono [91] studied the profitability and determinants of smallholder commercial vegetable production. They found that access to credit had the highest magnitude of marginal effects from their regression result, implying that it was an important determinant of crop production. Kassaw et al. [92] found that credit increased tomato supply in Ethiopia, while Onyeneke et al. [45] found that credit increased cassava, maize, and rice production in the long run in Nigeria. From a policy view point, credit creates a better outcome in agricultural production. Commercial herbaceous fruit crop production is capital-intensive and requires capital, which oftentimes may not be adequate or met through farmers’ savings only. Obtaining credit most times closes the agricultural financing gap experienced by farmers.

## 5. Conclusions, Policy Implications, and Recommendations

This study analyzed the impacts of climate change on herbaceous fruit crop (tomato and okra) yield in Nigeria using credit and fertilizer as covariates. Using time-series data spanning a period of 40 years and the ARDL model, we can conclude that climate change and agricultural production inputs have both short- and long-term impacts on okra and tomato yields. The impacts of climate and agricultural inputs on herbaceous fruit crops have revealed the significant roles temperature, fertilizer, and credit play in fruit crop growth and development.

Relying on long-term okra and tomato yield, climate, fertilizer consumption, and credit statistics obtained from the FAOSTAT, World Bank Climate Change Knowledge Portal, World Bank World Development Indicators, and Central Bank of Nigeria Statistical Bulletin, we observed that temperature has both short-term and long-term negative impacts on tomato and okra yields. This has implications on climate adaptation and planning. For policy purposes, we suggest that, to achieve SDG goal 1, for example, continued efforts are needed to review existing climate change policies, plans, and commitments that will promote investments in climate smart agriculture and dissemination of associated technologies to fruit crop farmers. This would reduce the risk of fruit crop failure whilst ameliorating the effect of climate change on the agricultural livelihoods of many people in Nigeria and Africa.

Moreover, we observed fertilizer consumption per hectare of arable land significantly decreased okra yield in the long run but insignificantly increased the yield in the short run. With this finding, policy actions should focus on precision agriculture and efficient use of fertilizer in fruit crop production. Therefore, mapping the soil fertility levels of different agroecological zones and localities in Nigeria and Africa would go a long way determining the exact quantity, quality, and type of fertilizer needed in each area and reduce overflow or underflow of nutrients in the soil. Therefore, we recommend the implementation or upscale of soil fertility testing projects in various localities on the continent.

Overall, this study strengthens the view that credit plays a significant role in crop production because of the positive long-term impact of credit on tomato yield. Therefore, making credit available to farmers will help to achieve the food and nutrition security and poverty reduction goals of the United Nations, Africa, and Nigeria. Thus, the study recommends that policies should be geared towards intensifying every effort to increase access to affordable credit facilities to farmers. Governments, financial institutions, and donors can formulate and implement sustainable and affordable credit access programs for fruit crop farmers.

One important limitation of this study needs to be mentioned. In general, maximum and minimum temperatures also have impacts on crop yield; this study only analyzed the effect of average temperature. Future studies should expand on this aspect by adding minimum and maximum temperatures in exploring the short- and long-run dynamics of climate change and crop production. The current study (and its robust design and adopted analytical techniques) nonetheless suffices for the illumination of the effects of climate change on herbaceous fruit crop production in Nigeria.

## Figures and Tables

**Table 1:** Data source.

Variables	Source
Okra yield (hg/ha)	FAOSTAT [67]
Tomato yield (hg/ha)	FAOSTAT [67]
Mean temperature (°C)	World Bank Climate Change Knowledge Portal [68]
Total rainfall (mm)	World Bank Climate Change Knowledge Portal [68]
Fertilizer consumption (kilograms per hectare of arable land)	World Development Indicators [69]
Total credit guaranteed to the crop sector (USD) ^a^	Central Bank of Nigeria Statistical Bulletin [70]

^a^ The official exchange rate data from Central Bank of Nigeria to convert Naira values of credit guaranteed to USD values to control for inflation.

**Table 2:** Descriptive statistics.

Variables	N	Minimum	Maximum	Mean	Standard Deviation	Skewness	Kurtosis
Okra yield (hg/ha)	40	8735.00	29,962.00	21,630.63	5797.07	−0.86	−0.27
Tomato yield (hg/ha)	40	37,208.00	103,455.00	76,881.20	22,429.92	−0.35	−1.28
Mean temperature (°C)	40	26.32	27.81	27.17	0.32	−0.33	0.35
Total rainfall (mm)	40	872.04	1269.15	1137.11	82.15	−0.82	1.22
Fertilizer consumption (kilograms per hectare of arable land)	40	4.15	20.97	10.04	4.76	0.71	−0.31
Total credit guaranteed to the crop sector (USD)	40	2201.43	60,838.61	17,196.85	14,694.91	1.20	0.56

**Table 3:** Multicollinearity statistics based on variance inflation factor (VIF).

Independent Variables	Variance Inflation Factor (VIF)
Mean Temperature (°C)	1.465
Total Rainfall (mm)	1.086
Fertilizer consumption (kilograms per hectare of arable land)	1.039
Total Credit to the Crop Sector (USD)	1.472

**Table 4:** Unit root test results for herbaceous fruit crops yield and climate change.

H_0_ = Series Have a Unit Root						
**Augmented Dickey–Fuller Test**						
	**At Level I(0)**	**Remark**	**At First Difference I(1)**	**Remark**	**Decision: H_0_**	**Order of Integration**
**Variable**	**t-Statistic**		**t-Statistic**			
Y1	−2.279 **	Stationary	−6.155 ***	Stationary	Reject	I(0) at 5%
Y2	−1.560	Not stationary	−6.617 ***	Stationary	Reject	I(1) at 1%
X1	−3.369 ***	Stationary	−8.612 ***	Stationary	Reject	I(0) at 1%
X2	−4.596 ***	Stationary	−8.880 ***	Stationary	Reject	I(0) at 1%
X3	−1.990 **	Stationary	−4.438 ***	Stationary	Reject	I(0) at 5%
X4	−1.750 **	Stationary	−4.325 ***	Stationary	Reject	I(0) at 1%
**Phillips–Perron Test**						
	**At Level I(0)**	**Remark**	**At First Difference I(1)**	**Remark**	**Decision: H_0_**	**Order of Integration**
**Variable**	**t-Statistic**		**t-Statistic**			
Y1	−3.019	Not stationary	−10.495 ***	Stationary	Reject	I(1) at 1%
Y2	−3.940 **	Not stationary	−6.377 ***	Stationary	Reject	I(0) at 5%
X1	−4.884 ***	Stationary	−8.664 ***	Stationary	Reject	I(0) at 1%
X2	−5.484 ***	Stationary	−12.347 ***	Stationary	Reject	I(0) at 1%
X3	−1.904	Not stationary	−8.452 ***	Stationary	Reject	I(1) at 1%
X4	−1.732	Not stationary	−6.119 ***	Stationary	Reject	I(1) at 1%

Note: ** and *** indicate significance at 5% and 1% levels, respectively.

**Table 5:** Results of ARDL-bounds cointegration test.

Tests	Null Hypothesis	Okra Yield	Tomato Yield
Ramsey RESET test	Model has no omitted variables	0.24 (0.8701)	1.18 (0.33)
White’s test	Homoskedasticity	20.82 (0.11)	18.37 (0.19)
F test	No levels relationship	5.077 ***	3.504 *

Note: *p*-values are in parentheses; *** denotes statistical significance at 1%; * denotes statistical significance at 10%.

**Table 6:** ARDL estimates for the impact of climate change on herbaceous fruit crop yield.

	Okra Yield	Tomato Yield
**Long-run estimates**		
lnX1	−28.96	−35.81
	(−3.01) ***	(−4.66) ***
lnX2	1.40	−0.856
	(1.24)	(−1.13)
lnX3	−0.48	−0.04
	(−3.26) ***	(−0.38)
lnX4	0.05	0.15
	(0.54)	(1.78) *
**Short-run estimates**		
ECM	−0.53	−0.50
	(−3.61) ***	(−3.92) ***
ΔlnX1	−6.92	−6.67
	(−1.89) *	(−2.09) **
ΔlnX1(-1)		11.41
		(3.71) ***
ΔlnX2	0.74	−0.43
	(1.46)	(−1.10)
ΔlnX3	0.08	−0.02
	(0.71)	(−0.37)
ΔlnX4	0.03	0.07
	(0.54)	(1.76) *
Constant	50.93	67.07
	(3.39) ***	(3.75) ***
R^2^	0.50	0.41

Note: z-values are presented in parenthesis. *** denotes statistical significance at 1%, ** denotes statistical significance at 5%, and * denotes statistical significance at 10%. ECM is the error correction term/speed of adjustment towards the long-run equilibrium.

## Data Availability

The data used for this study are openly/publicly available. The data can be downloaded from the World Development Indicators website, World Bank Climate Change Knowledge Portal website, FAOSTAT website, and Central Bank of Nigeria website.
